# What shall I be, what must I be: neural correlates of personal goal activation

**DOI:** 10.3389/fnint.2012.00123

**Published:** 2013-01-07

**Authors:** Timothy J. Strauman, Allison M. Detloff, Rima Sestokas, David V. Smith, Elena L. Goetz, Christine Rivera, Lori Kwapil

**Affiliations:** Department of Psychology and Neuroscience, Duke UniversityDurham, NC, USA

**Keywords:** regulatory focus, promotion, prevention, personal goals, fMRI, behavioral activation system, behavioral inhibition system

## Abstract

How is the brain engaged when people are thinking about their hopes, dreams, and obligations? Regulatory focus theory postulates two classes of personal goals and motivational systems for pursuing them. Ideal goals, such as hopes and aspirations, are pursued via the *promotion* system through “making good things happen.” Ought goals, such as obligations or responsibilities, are pursued via the *prevention* system through “keeping bad things from happening.” This study investigated the neural correlates of ideal and ought goal priming using an event-related fMRI design with rapid masked stimulus presentations. We exposed participants to their self-identified ideal and ought goals, yoked-control words and non-words. We also examined correlations between goal-related activation and measures of regulatory focus, behavioral activation/inhibition, and negative affect. Ideal priming led to activation in frontal and occipital regions as well as caudate and thalamus, whereas prevention goal priming was associated with activation in precuneus and posterior cingulate cortex. Individual differences in dysphoric/anxious affect and regulatory focus, but not differences in BAS/BIS strength, were predictive of differential activation in response to goal priming. The regions activated in response to ideal and ought goal priming broadly map onto the cortical midline network that has been shown to index processing of self-referential stimuli. Individual differences in regulatory focus and negative affect impact this network and appeared to influence the strength and accessibility of the promotion and prevention systems. The results support a fundamental distinction between promotion and prevention and extend our understanding of how personal goals influence behavior.

## Introduction

A person's hopes, dreams, and wishes, whether attained or unattained, have always been seen as central to an individual's identity—as the essence of who a person is because they represent that which a person strives to be (James, 1890/1948). The kind of individual we wish to be, and the kind of person we believe we must be, are powerful influences on behavior and affect whether we see ourselves as succeeding or failing to attain those wishes and obligations (Kelly, 1955). Classic (Allport, 1955) as well as contemporary (Morf and Mischel, 2012) personality theorists, for example, have used the concept of *becoming* as a rubric for understanding individual differences in motivational orientation along with the affective consequences of failing to be that which we want to be or must be. Personal goals are real in a profoundly psychological sense—dreams and obligations are “truth” for individuals whether or not they “come true.”

The *goal* construct in psychology captures much of how hopes, dreams, and wishes guide behavior and experience. Behavioral scientists have conceptualized personality as reflecting differences among people in terms of the higher-order goals they pursue and their characteristic ways of pursuing them (Cantor and Zirkel, 1990). Indeed, goals are a central construct in theories of behavior because they provide a unified conceptual framework linking internal states (needs, motives, beliefs) and the social world. When people believe they have attained an important goal, they may feel joyful, satisfied, fulfilled, or worthy; when people believe they have failed to attain a goal, they may feel inadequate, hopeless, worthless, guilty, or ashamed (Sullivan, 1953; Rogers, 1961). Yet, to date there has been little research examining how neural systems are engaged when people think about their personal goals, both when the goals are attained as well as when they are not.

In an influential review, Austin and Vancouver (1996) defined goals as *internal representations of desired states* and identified approach and avoidance goals as among the most important classes of goals. The vast behavioral science and neuroscience literatures on approach and avoidance attest to the centrality of these dimensions for understanding goal-directed behavior. Those literatures are dominated by the behavioral activation and inhibition systems model, postulating brain/behavior systems that underlie temperament-based approach and avoidance as well as dispositional positive and negative affectivity (Watson et al., 1999). Both the behavioral activation system (BAS) and behavioral inhibition system (BIS) are hypothesized to regulate proximal goal-directed behaviors in response to cues for reward, in the case of BAS, or threat, in the case of BIS (Carver and Scheier, 1998). The two systems each represent a locus for the interaction of cognitive and affective processes, and each is associated with neural circuitry identified originally on the basis of animal research and shown to have analogs in the human brain (Gray, 1990, 1994).

Many personality and social theorists take a complementary perspective on the regulation of approach and avoidance, emphasizing abstract, higher-order goals that are cross-situational and integrated within the individual's sense of self. Higgins (1997) proposed a theory of *regulatory focus* that postulated two motivational systems for attainment of desired outcomes. Each is activated by contextual cues but also manifests trait-like properties across situations. Individual differences in regulatory focus are stable over time and predict which goals will be more likely to be used to guide behavior, as well as the strategies and means for pursuing them (Strauman, 1996). The behavioral and affective consequences of individual differences in regulatory focus are well-established (Higgins, 2012).

RFT draws on prior studies of self-evaluation and discrepancy monitoring and describes two regulatory systems that serve critical but distinct survival needs. The *promotion system*, which develops in response to children's need for nurturance (Bowlby, 1988), supports the attainment of positive outcomes by strategic approach, i.e., by “making good things happen.” The promotion system is particularly active in the pursuit of ideals (aspirations, advancement, and accomplishment)—that is, the kind of person an individual can be or might be. The *prevention system*, which develops in response to children's need for security (Bowlby, 1988), also supports the attainment of positive outcomes, but instead by strategic avoidance, i.e., by “keeping bad things from happening.” The prevention system is particularly active in pursuit of oughts (fulfillment of responsibilities, duties, and obligations)—that is, the kind of person an individual believes she/he must be or is supposed to be.

Individuals vary both in the characteristic ways they construe their goals and their chosen strategies to pursue them. As a consequence of variation in life experiences, a person might acquire increased value or personal relevance for one type of goal. For example, a strong value placed on prevention goals will result in goal pursuit strategies that involve keeping bad things from happening—for example, by avoiding pitfalls and negative outcomes in the service of ultimate goal attainment. In addition, the same desired end-state can be represented in different ways by prevention-oriented vs. promotion-oriented individuals. The same goal—such as being honest—could be represented as an ideal or aspiration (a promotion goal) or as an obligation or responsibility (a prevention goal).

In this article, we examine the neural correlates of priming personal goals using ideals and oughts as exemplars of the two types of goal representations postulated within RFT. We consider three largely unexplored questions about how the brain is engaged in pursuit of ideals and oughts. First, does priming of an individual's ideal vs. ought goals, already shown to result in distinct cognitive, motivational, and affective responses, also lead to discriminable patterns of neural activation? Second, do activation patterns associated with ideal vs. ought goals vary as a function of individual differences relevant to self-regulation? And third, since self-regulatory cognition is inherently connected with affect and with vulnerability to disorders such as depression and anxiety, do activation patterns observed following ideal and/or ought goal priming vary depending on an individual's current level of negative affect?

Based on existing findings in social cognitive neuroscience, there are several different patterns of neural activation that might characterize responses to priming of promotion and prevention goals (and, in turn, contribute to the construct validity of regulatory focus). One set of regions are those structures known to be activated by reward or threat cues, consistent with the role of BAS and BIS as mechanisms for individual differences in sensitivity to such cues (Amodio et al., 2007). There are conceptual links between the BAS system and the promotion system, since promotion goal pursuit requires responsiveness to opportunities for rewards in the environment and the use of strategic approach behaviors to achieve desired ends. There are similar links between the BIS system and the prevention system, because of the relevance of the strategic avoidance of negative outcomes in prevention goal pursuit. BAS-related regions implicated in response to incentives include the ventral striatum and ventromedial prefrontal cortex, with the former a locus for the coding of predictions regarding positive outcomes and the latter important for the processing of the hedonic significance of stimuli (Bjork et al., 2004; McClure et al., 2004; Kringelbach, 2005; Clithero et al., 2011). Individual differences in BIS strength have been associated with circuits linking the hippocampus, subiculum, and related structures (sometimes also including the basolateral and centromedial nuclei of the amygdala) (e.g., Reuter et al., 2004). Thus, the neural correlates of promotion vs. prevention could reflect the neuroanatomical distinctions between the substrates of BAS and BIS.

Another potential set of neural correlates of promotion/prevention goal activation is the group of regions referred to collectively as cortical midline structures (Northoff and Bermpohl, 2004; Lou et al., 2010; Qin and Northoff, 2011). These structures, which typically include the orbital and adjacent medial prefrontal cortex, the anterior cingulate cortex, the dorsomedial prefrontal cortex, and the posterior cingulate cortex, are regarded as an anatomical unit because of strong reciprocal projections among the individual structures and similar patterns of connectivity with other brain regions. They also are characterized as a network that subserves the representation and processing of self-referential stimuli (Beer and Ochsner, 2006). This set of regions may underlie the activation of promotion and prevention goals that are functionally linked to aspects of one's identity, including higher-order goals representing one's ideal self or ought self.

A third possibility is that the promotion and prevention goal representations overlap with the Self-Memory System postulated by Conway (SMS; Conway and Pleydell-Pierce, 2000). The SMS is a conceptual framework linking self and memory that consists of two main components: the working self and the autobiographical memory knowledge base. Drawing in part on studies of self-discrepancy and autobiographical memory (e.g., Strauman, 1990), Conway (2001) proposed that frontotemporal networks mediate the connection between anterior regions associated with the working self (e.g., one's currently active goals and beliefs) and the autobiographical knowledge base, accessed through temporal lobe regions, needed to effectively pursue such goals within a dynamic interpersonal context.

Several studies have examined associations between regulatory focus and brain activity, with the evidence to date suggesting a link between promotion/prevention and midline cortical structures as well as a pattern of prefrontal cortex asymmetry akin to that observed in the BAS/BIS literature (Davidson and Irwin, 1999). Amodio et al. (2004) examined the associations between an implicit assessment of individual differences in regulatory focus and an EEG index of resting frontal cortical asymmetry. They observed that chronic promotion focus was associated with greater left frontal activity, whereas chronic prevention focus was associated with greater right frontal activity. Cunningham et al. (2005) found that neural activation when making good/bad judgments differed by individuals' regulatory focus: chronic promotion focus was associated with greater activation in the amygdala, anterior cingulate, and extrastriate cortex following positive stimuli, and chronic prevention focus was associated with activity in the same regions for negative stimuli. Touryan et al. (2007) also used fMRI to study the impact of individual differences in regulatory focus on memory for emotional words. They observed that activity in posterior cingulate cortex, associated with self-referential processing, was greater for correctly remembered stimulus words when they were consistent with an individual's regulatory focus. Packer and Cunningham (2009) investigated how regulatory focus interacted with reflection on personal goals and observed differential activation patterns according to goal domain (promotion vs. prevention) and temporal distance (short-term vs. longer-term).

Two studies have used idiographically selected promotion and prevention goals as stimuli within fMRI designs. Eddington et al. (2007) used incidental semantic priming via a “depth of processing” judgment task to examine patterns of cortical activation associated with promotion and prevention goals. An area of left PFC was activated during promotion goal priming across all four judgment tasks, and the magnitude of activation in this region was correlated significantly with individual differences in strength of orientation to promotion goals. In contrast, activation at this site did not correlate significantly with orientation to prevention goals or with individual differences in BAS/BIS strength. Eddington et al. (2009) examined the neural correlates of promotion and prevention goal priming in a sample of unmedicated adult patients meeting Diagnostic and Statistical Manual-IV-R criteria for major depressive disorder (MDD) as well as an age- and gender-matched control sample of adults with no psychiatric history, using the same judgment task. They hypothesized that MDD patients would show an attenuated left PFC response to promotion priming compared to the non-depressed controls. There was a significant difference in activation between the depressed and non-depressed groups following promotion goal priming, with controls showing greater left medial orbital PFC activation following promotion priming than the depressed patients. In addition, a region in right PFC was activated following prevention priming among MDD patients with comorbid anxiety.

The findings to date suggest that promotion and prevention may be associated with distinct patterns of neural activation, but none of the prior studies was designed specifically to address that question. In the present study, we adapted an fMRI paradigm developed by Diaz and McCarthy (2007) for rapid masked presentation of semantic stimuli. Masking provides a method for identifying cognitive processes that are preattentive, routinized, and automatic (Dehaene et al., 2001). The use of rapid masked idiographic goal priming offers several significant advantages. First, promotion and prevention goals can be activated automatically like other highly accessible social constructs (i.e., without intentional selection of a goal upon which to focus one's efforts); therefore, a paradigm that would allow detection of implicit priming effects was highly desirable. Second, the two studies by Eddington and colleagues were restricted in the number of goal priming trials included because individuals typically describe a small number of motivationally significant personal goals, thereby limiting the number of goal words available for use as explicit priming stimuli. The use of rapid masked stimulus presentation allows for a greater number of trials within an event-related design (in part because stimuli can be repeated more frequently). Third, because the participant's task in the Diaz and McCarthy paradigm is simply to make a response whenever she/he sees a non-word stimulus in color (e.g., ampersands in red font), a task that was non-self-referential, there is less potential for overlap or interference between the experimental task and priming-based activation of idiographically selected promotion and prevention goals.

Using this paradigm, we explored three aspects of the neural correlates of promotion and prevention goal representations. First, we examined whether BOLD activation patterns would differ for idiographic priming of promotion goals vs. prevention goals. Second, we examined whether activation in regions associated with promotion/prevention goal priming would be correlated with ratings of perceived success pursuing goals and/or BAS/BIS strength. Third, we examined whether the activation patterns observed following promotion and/or prevention goal priming would be modulated by the individual's current level of negative affect, specifically dysphoric and anxious symptoms.

## Materials and methods

### Overview

Based on an event-related fMRI paradigm developed by Diaz and McCarthy (2007), participants were exposed to a continuing series of rapidly presented masked visual stimuli including (1) a subset of each participant's ideal and ought goals assessed in a prior session, (2) ideal and ought goals of a different participant (as a yoked-control condition), and (3) non-word letter strings. Participants were told that the task was to respond as quickly as possible whenever they detected a string of letters or symbols presented in a colored font. The detection task was actually a means to keep participants attending to the continuous stimulus presentation. BOLD signal responses to ideal and ought goals were contrasted with responses to control words to test hypotheses about neural correlates of personal goal activation.

### Participants

Participants were recruited through the introductory psychology research pool at Duke University and were part of a larger sample (*N* = 75) who had completed a study earlier in the semester. The initial study session, described as an investigation of personality, included several self-report measures relevant to the present research. Approximately two months after the personality study, potential subjects were contacted by phone and invited to participate in what was described as an investigation of visual attention. Thirty-three students (16 male) agreed to participate; one withdrew from the study prior to the MRI session for medical reasons, and a second student's imaging data were unusable due to technical problems; thus, data from 31 participants were included in analyses. All participants were between the ages of 18 and 22 and were right-handed as indicated by self-report. Participants reported normal neurological history and had normal or corrected-to-normal visual acuity. All participants gave informed consent in accordance with Duke University Institutional Review Board guidelines and received cash payment as compensation for their time.

### Procedure

#### Individual difference measures

During the personality study session, participants completed a measure of chronic regulatory focus, a measure of temperament-based approach and avoidance tendencies, and two measures of distress. The *Regulatory Focus Questionnaire* (RFQ; Higgins et al., 2001) is a 22-item Likert-style instrument designed to measure individual differences in orientation toward promotion and prevention goals. The RFQ contains four scales (two each for promotion and prevention): two *history* scales measuring the extent to which the individual's socialization history was characterized by an emphasis on promotion or prevention goals, and two *success* scales measuring the extent to which the individual believes she/he has been successful in attaining promotion or prevention goals. Because the psychometric properties of the history scales have yet to be determined, only the success scales were used in the present study. Sample items include: “I feel like I have made progress toward being successful in my life” (promotion success); and “Not being careful enough has gotten me into trouble at times” (prevention success—reverse-scored). Higgins et al. (2001) reported that the success scales had internal consistency reliability (coefficient alpha) of 0.75 or higher, and a 2-month test-retest reliability (Pearson correlation) of 0.79 or higher.

The *BIS/BAS Scale* (BIS/BAS; Carver and White, 1994) is a well-validated instrument containing four scales to measure individual differences in BAS and BIS sensitivity: BIS subscale (coefficient alpha = 0.74), BAS reward responsiveness subscale (coefficient alpha = 0.73), BAS drive subscale (coefficient alpha = 0.76), and BAS fun-seeking subscale (coefficient alpha = 0.66). We report results for a general BAS principal component score combining all three BAS subscales.

The *Beck Depression Inventory* (BDI; Beck et al., 1961) is a widely used 21-item measure of depressive and dysphoric symptoms. Respondents are asked to endorse items varying in severity from (0) to (3) in a number of life areas. For example, “I do not feel sad” scored (0); and “I am so sad or unhappy that I can't stand it” scored (3). The highest rating for each item was summed across all 21 items to create a continuous measure of depressive symptoms.

The *State Trait Anxiety Inventory* (STAI; Spielberger et al., 1970) is a self-report assessment which includes separate measures of state anxiety and the more general quality of trait anxiety. Participants completed only the trait version for the current investigation. The essential qualities evaluated by the 20-item STAI-T scale are feelings of apprehension, tension, nervousness, and worry (e.g., “I am jittery”). Items are rated on a 4-point scale ranging from 1 (*not at all*) to 4 (*very much so*) and summed to create a scale score. Given the high correlations for the BDI and STAI in this sample (*r* = 0.75), and our anticipation that we would not find distinct patterns of dysphoric vs. anxious affect in this unselected sample of healthy college students (Nitschke et al., 2001), the two scales were combined by summing individual scale z-scores into a single dysphoric/anxious index representing current level of negative affect.

#### Promotion and prevention goal generation and selection

During the personality study session, participants also completed a computerized version of the *Selves Questionnaire* (SQ; Higgins et al., 1986). The SQ is a semi-structured measure that was used to sample participants' own promotion and prevention goals. Participants listed traits or attributes for two different self-state representations: the attributes of the kind of person they ideally would like to be (ideal self-guides, which function as promotion goals) and the attributes of the kind of person they believe it is their obligation or responsibility to be (ought self-guides, which function as prevention goals). Personal goal stimuli for the priming task were obtained from each participant's responses to the computerized SQ. Following the procedures used by Strauman (1996), four promotion goals (“ideal self” responses) and four prevention goals (“ought self” responses) that were semantically unrelated were identified for each participant from among that participant's total set of SQ responses. All of the goals selected from participants' SQ responses were positively valenced. Then the promotion and prevention goals were pooled across subjects, and for each participant a set of eight yoked-control words was selected from that pool so that each yoked-control word was semantically unrelated to all of the participant's promotion and prevention goals. The yoked-control priming condition was included to rule out the alternative hypothesis that the semantic content of an ideal or ought word, rather than its status as a personal goal, might account for the activation observed following a priming trial.

#### Goal priming task

The fMRI task was adapted from Diaz and McCarthy (2007). Participants viewed a continuous stream of masked words and non-words while performing a detection task in which they were asked to make a response to a visible colored non-word stimulus (e.g., percent signs in red font). The detection task ensured participant engagement, while the non-word priming controlled for perceptual and orthographic processing. The masked word stimuli were of three types: a subset of the participant's promotion (ideal) goals, a subset of the participant's prevention (ought) goals, and yoked-control words (ideal and ought goals of other participants which had no self-regulatory significance for the participant her/himself). Three runs, each 600 s in length, were conducted. The stimuli for each run consisted of words (ideal goals, ought goals, or a yoked-control word) and non-word letter strings that were masked and displayed in a fixed width font. All goal words (ideal, ought, and yoked-control) were positively valenced trait attributes. The non-words were random consonant strings, each 4–10 characters in length. Each letter string was padded with pound signs so that letters were centered and each stimulus was 12 characters in length, in order to ensure that the same amount of the visual field was occupied on any given priming trial.

**Figure 1:**
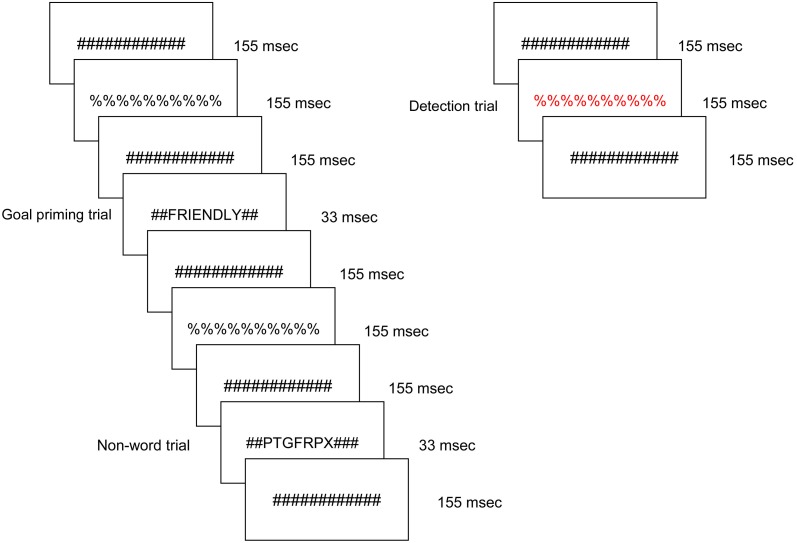
**Schematic of the experimental task, displaying a typical sequence of priming trials.** The sequence for an individual trial consisted of alternating pound signs and percent signs, in between which a word or non-word was inserted. Promotion goal, prevention goal, and yoked-control priming stimuli were inserted throughout the run. Incidental to those stimuli visible colored symbol stimuli were displayed to which participants were instructed to respond with a button press as quickly as possible.

Figure 1 presents an example sequence of priming trials within the overall experimental design as viewed by each participant. Participants viewed a constantly changing visual display in which a stimulus was presented every 1500 ms for a duration of 33 ms. The majority of the trials were masked non-words; a masked word from one of the three remaining stimulus conditions was presented approximately every 12 s. In each run, 16 ideal priming trials, 16 ought priming trials, and 16 yoked-control priming trials were included. All word and non-word letter strings were preceded and followed by pound sign strings for 155 ms, which served as pattern masks. In turn, the pound sign strings alternated with percent sign strings such that the subject was exposed to a continuously changing visual stream. The masked non-word trials ensured that brain regions responsive to physical features and orthography would be continuously active. Any brain region that was responsive to one of the three word priming conditions therefore reflected a higher level of cognitive processing. Participants were instructed to press a button when they detected a letter or symbol string presented in a color font. Those target events occurred infrequently (mean interval = 25 s) and were not in close temporal proximity to word priming trials. All stimuli were displayed on MRI-compatible LCD goggles.

#### Manipulation check

The participants were not informed that words or non-words would be presented. In order to evaluate subjects' perceptions of the masked stimuli, both subjective and objective assessments were conducted. Prior to imaging, participants were shown individual masked trials and were asked to report “anything and everything that you see.” No participant reported seeing words, and most simply reported that they saw rapidly flashing strings of pound signs or percent signs. After the three runs were completed, subjects again were questioned about what they experienced and then completed a brief questionnaire in which both words that had been presented in each of the three word priming conditions and an equal number of words that had not been presented were listed. Participants were told that during the task they had been exposed periodically to words and were asked to indicate whether they believed each word had been presented or not. No participant performed significantly beyond chance levels in identifying presented vs. not-presented words.

#### fMRI parameters and data processing

Functional images sensitive to blood oxygenation level-dependent (BOLD) contrast were acquired using an inverse spiral pulse sequence (TR, 1.5 s; TE, 35 ms; FOV, 24 cm; image matrix, 64^2^; 34 contiguous axial slices; voxel size 3.75 × 3.75 × 3.8 mm) on the research-dedicated 3.0 Tesla GE Signa EXCITE HD system at Duke's Brain Imaging and Analysis Center (BIAC: www.biac.duke.edu). The 3.0 T has an eight-channel head coil for parallel imaging at high bandwidth up to 1 MHz, in addition to its volume birdcage head coil. Each of the three runs consisted of the acquisition of a time series of 242 brain volumes (TR = 1.5 s, run length = 363 s). Four initial RF excitations were performed (and discarded) to achieve steady state equilibrium. High-resolution structural images were acquired using a 3D fast SPGR pulse sequence (TR, 12.2 ms; TE, 5.3 ms; FOV, 24 cm; image matrix, 256^2^; voxel size 0.9375 × 0.9375 × 1.9 mm). A semi-automated high-order shimming program was used to ensure global field homogeneity.

Analyses of the BOLD signal were conducted using FEAT (FMRI Expert Analysis Tool; Smith et al., 2004; Woolrich et al., 2009), part of FSL (FMRIB's Software Library, Oxford University; www.fmrib.ox.ac.uk/fsl). The following pre-statistics processing steps were applied: motion correction using MCFLIRT, slice-timing correction, removal of non-brain voxels using BET, spatial smoothing with a Gaussian kernel of FWHM 8 mm, and high-pass temporal filtering with a cutoff of 100 s. Registration to high resolution and standard images was carried out using FLIRT.

#### Statistical analyses

Analyses were conducted to identify regions reliably activated by ideal and ought goal priming respectively, and each proceeded in three stages. First, preprocessed functional data were analyzed using a general linear model with local autocorrelation correction (Woolrich et al., 2001). For each run, we set up separate regressors for promotion (ideal), prevention (ought), yoked-control, and non-word primes. A nuisance regressor modeled the target detection component of the task. All regressors consisted of unit impulses convolved with a canonical hemodynamic response function. The contrast of interests were comparing ideal vs. control and ought vs. control priming. We then combined data across runs for each subject using a fixed-effects model, and combined data across subjects using a mixed-effects model (Beckmann et al., 2003; Woolrich et al., 2004). We also used the mixed-effects models to obtain statistical tests for whether the two individual differences measures of interest (promotion/prevention success and BAS/BIS strength) and the dysphoric/anxious symptom index significantly modulated activation following goal priming. All *z*-statistic (Gaussianised *t*) images were thresholded using clusters determined by *z* > 2.3 and a corrected cluster-significance threshold of *p* < 0.05 (Worsley, 2001). As Brodmann labels can be somewhat misleading (Zilles and Amunts, 2010), we report probabilistic anatomical labels for local maxima within statistically significant clusters derived from the Harvard-Oxford Cortical and Subcortical Structural Atlases along with approximate Brodmann areas.

## Results

### BOLD activation: promotion > control priming

**Figure 2:**
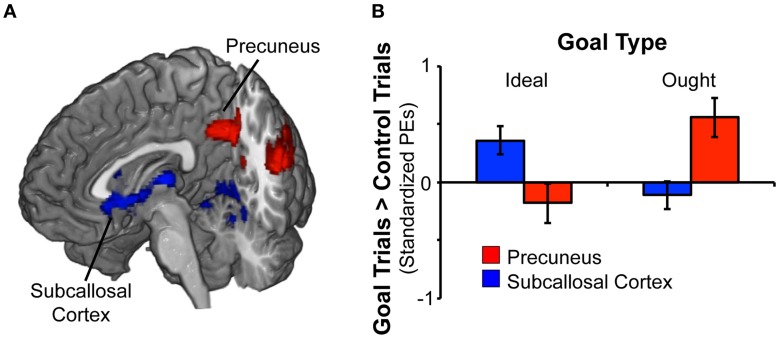
**Rapid masked promotion and prevention goal priming induced distinct patterns of activation. (A)** To identify brain regions associated with activation of a promotion goal, we contrasted ideal goal priming vs. control priming. There was a significant effect for promotion goal priming in both subcallosal cortex and lingual gyrus (shown in blue). Likewise, to identify brain regions associated with activation of a prevention goal, we contrasted ought goal priming vs. control priming. There was a significant effect for prevention goal priming in precuneus and posterior cingulate gyrus (shown in red). **(B)** The chart displays mean standardized parameter estimates (PEs) at selected sites for the ideal vs. control contrast as well as the ought vs. control contrast. Within the subcallosal cortex (*x* − 10, *y*26, *z* − 10), activation was significant greater in response to ideal priming (vs. control priming). Within the precuneus (*x* − 32, *y*14, *z*46), activation was significantly greater in response to ought priming (vs. control priming).

We analyzed the fMRI data for promotion goal priming by contrasting responses to ideal priming with responses to yoked-control priming. As shown in Table 1; Figure 2, we found two brain regions that responded significantly more in response to ideal primes than to yoked-control primes, constituting main effects for promotion priming. The first cluster included occipital pole and lingual gyrus (both bilateral, approximately BA 18). The second cluster, predominantly left-sided, included subcallosal cortex (approximately BA 11/25), caudate, and thalamus.

**Table 1:** **Regions showing significantly greater activation following promotion (ideal) priming trials compared to control priming trials**.

**Probabilistic anatomical label**	***x***	***y***	***z***	***Z* statistic**	**Cluster volume (*p*)**
**MAIN EFFECT FOR CONTRAST**					
Occipital pole (57%), supercalcarine cortex (6%)	4	−94	18	3.89	2451 mm^3^ (*p* < 0.01)
Lingual gyrus (70%)	2	−80	−6	3.41	
Lingual gyrus (50%)	−8	−66	−8	3.29	
Lingual gyrus (17%)	−6	−70	−12	3.10	
Lingual gyrus (69%), intracalcarine cortex (16%)	2	−74	2	3.08	
Lingual gyrus (5%)	0	−68	−8	3.00	
Paracingulate gyrus (10%)	−14	44	−4	3.90	1677 mm^3^ (*p* < 0.05)
Subcallosal cortex (14%)	−12	26	−4	3.78	
Subcallosal cortex (60%)	0	12	−4	3.70	
Thalamus (98%)	4	−18	8	3.68	
Subcallosal cortex (54%)	−10	26	−10	3.53	
Subcallosal cortex (45%)	−6	24	−4	3.48	
**POSITIVELY CORRELATED WITH PROMOTION SUCCESS SCORE**					
Precuneus cortex (41%), cuneal cortex (9%)	−8	−74	36	4.02	2878 mm^3^ (*p* < 0.01)
Posterior cingulate gyrus (37%)	−10	−34	38	3.95	
Precuneus cortex (42%), posterior cingulate gyrus (42%)	−2	−54	18	3.35	
Posterior cingulate gyrus (72%), anterior cingulate gyrus (21%)	0	−18	40	3.16	
Precuneus cortex (51%), posterior cingulate gyrus (6%)	6	−56	16	3.14	
Superior parietal lobule (8%), angular gyrus (6%)	−30	−52	34	3.09	
Right caudate (93%)	10	16	6	3.71	2138 mm^3^ (*p* < 0.01)
Left caudate (45%)	−24	−28	12	3.45	
Left thalamus (38%)	−2	−26	6	3.34	
Right thalamus (100%)	6	−16	10	3.27	
Left thalamus (88%)	−4	−24	2	3.10	

Coordinates of local maxima within each cluster of activation are in MNI space. Probabilistic labels reflect the likelihood that a coordinate belongs to a given region; for clarity, only labels whose likelihood exceeds 5% are shown.

**Figure 3:**
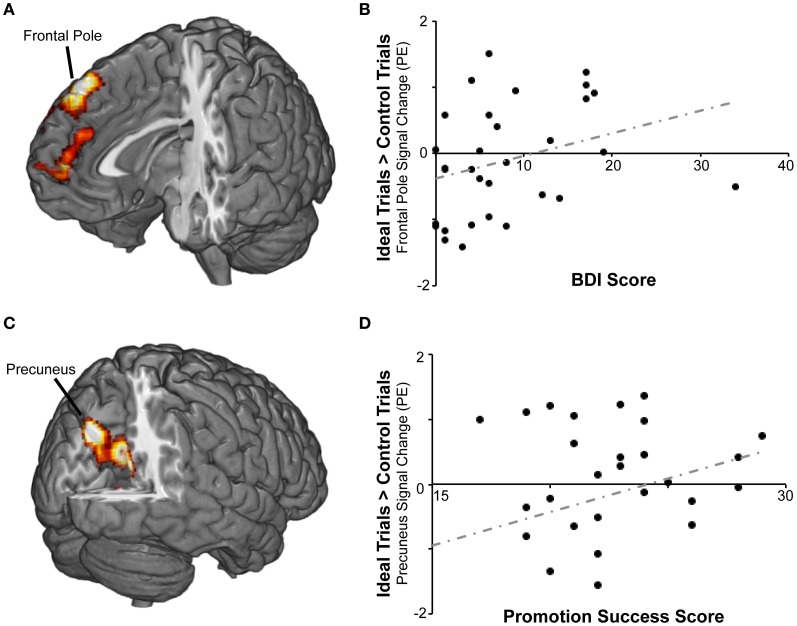
**BOLD responses to promotion goal priming were modulated by individual differences in self-reported success pursuing promotion goals and by scores on the symptom index. (A)** For the ideal > control contrast, symptom index scores were positively correlated with activation in frontal pole and paracingulate gyrus. **(B)** The scatterplot displays mean standardized PEs at the right frontal pole (*x*8, *y*40, *z*52) as a function of symptom index scores. **(C)** For the ideal > control contrast, promotion success scores were positively correlated with activation in precuneus, posterior cingulate, and other regions. **(D)** The scatterplot displays mean standardized PEs at the left precuneus (*x* − 8, *y* − 74, *z*36) as a function of promotion success scores.

We then repeated the analysis including covariates representing individual differences in self-regulation, BAS/BIS, and current level of dysphoric/anxious symptoms (see below) to determine whether responses to ideal priming were modulated by any of these variables. There were no significant findings for BAS or BIS strength, but we found two areas in which activation for ideal primes compared to yoked-control primes increased as individuals reported *higher* levels of success attaining *promotion* goals (Table 2; Figure 3). The first cluster included bilateral precuneus cortex (approximately BA 7) and bilateral posterior and anterior cingulate cortex (approximately BA 23 and 31). The second cluster included bilateral caudate and thalamus.

**Table 2:** **Regions showing significantly greater activation following prevention (ought) priming trials compared to control priming trials**.

**Probabilistic anatomical label**	***x***	***y***	***z***	***Z* statistic**	**Cluster volume (*p*)**
**MAIN EFFECT FOR CONTRAST**					
Precuneus cortex (62%), posterior cingulate gyrus (13%)	–6	–54	38	3.77	1815 mm^3^ (*p* < 0.05)
Precuneus cortex (90%)	4	–58	36	3.65	
Precuneus cortex (44%)	8	–60	36	3.64	
Posterior cingulate gyrus (24%)	20	–36	26	3.42	
Posterior cingulate gyrus (14%)	–24	–36	30	3.08	
**POSITIVELY CORRELATED WITH PREVENTION SUCCESS SCORE**					
Lateral occipital cortex (68%)	36	–70	54	3.72	1579 mm^3^ (*p* < 0.05)
Angular gyrus (26%), superior parietal lobule (10%)	36	–54	38	3.59	
Lateral occipital cortex (12%)	36	–76	56	3.55	
Precuneus cortex (16%)	20	–44	40	3.34	
Superior parietal lobule (15%)	26	–48	42	3.27	
Precuneus cortex (17%)	16	–56	34	3.19	
**NEGATIVELY CORRELATED WITH PROMOTION SUCCESS SCORE**					
Middle frontal gyrus (17%)	–32	14	46	3.75	1614 mm^3^ (*p* < 0.05)
Superior frontal gyrus (43%)	–20	36	48	3.45	
Superior frontal gyrus (48%)	–14	26	62	3.37	
Superior frontal gyrus (20%), Middle frontal gyrus (7%)	–26	18	64	3.33	
Superior frontal gyrus (20%)	–16	24	50	3.30	
Frontal pole (63%), superior frontal gyrus (8%)	–12	44	44	3.29	

Coordinates of local maxima within each cluster of activation are in MNI space. Probabilistic labels reflect the likelihood that a coordinate belongs to a given region; for clarity, only labels whose likelihood exceeds 5% are shown.

### BOLD activation: prevention > control priming

We analyzed the fMRI data for prevention goal priming by contrasting responses to ought priming with responses to yoked-control priming. As shown in Table 2; Figure 2, we found a cluster comprised of two subregions constituting main effects of prevention priming that responded significantly more to ought primes than to yoked-control primes: left and right precuneus cortex (approximately BA 7) and left and right posterior cingulate gyrus (approximately BA 31).

**Figure 4:**
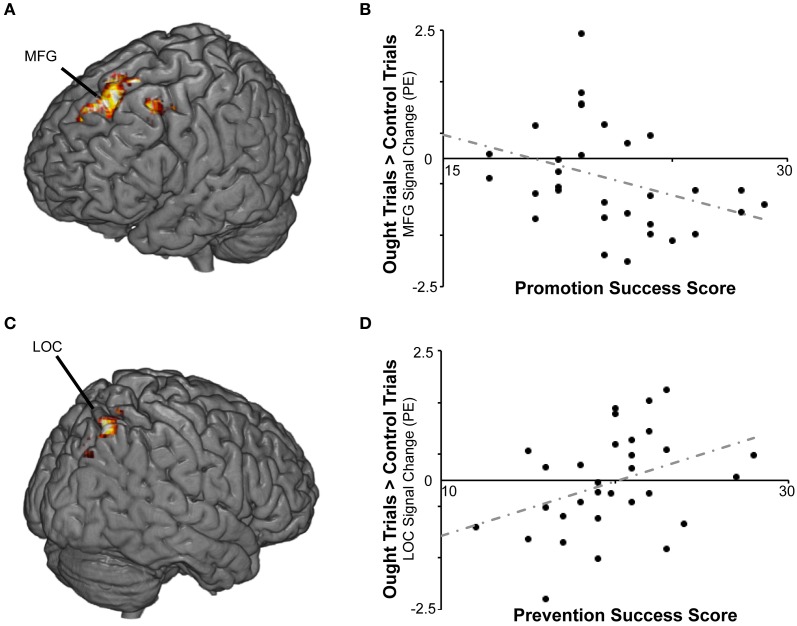
**BOLD responses to prevention goal priming were modulated by individual differences in self-reported success pursuing both types of personal goals. (A)** For the ought > control contrast, promotion success scores were negatively correlated with activation in middle (MFG) and superior frontal gyrus. **(B)** The scatterplot displays mean standardized PEs at the left middle frontal gyrus (*x* − 32, *y*14, *z*46) as a function of promotion success scores. **(C)** For the ought > control contrast, prevention success scores were positively correlated with activation in lateral occipital cortex (LOC) and other regions. **(D)** The scatterplot displays mean standardized PEs at the right LOC (*x*36, *y* − 70, *z*54) as a function of prevention success scores.

We then repeated the analysis including the covariates described above. Again there were no significant findings for BAS or BIS strength, but we found a cluster in which response to ought primes relative to yoked-control primes increased as individuals reported *higher* levels of success attaining *prevention* goals (Table 2; Figure 4). That cluster, which was entirely right-sided, included lateral occipital cortex (approximately BA 7), angular gyrus (approximately BA 40), precuneus cortex (approximately BA 31), and superior parietal lobule (also approximately BA 7). We also found a cluster in which response to ought primes relative to yoked-control primes increased as individuals reported *lower* levels of success attaining *promotion* goals. That cluster, which was entirely left-sided, included superior frontal gyrus (approximately BA 8) and middle frontal gyrus (approximately BA 6).

### Goal priming BOLD responses modulation by negative affect

In order to determine whether responses to ideal priming were modulated by current distress level, we also included the dysphoric/anxious index within the ideal vs. control contrast analysis as a covariate. As shown in Table 3, we found a cluster that showed a significantly greater response to ideal primes than to yoked-control primes as individuals reported higher levels of negative affect. This bilateral cluster included the frontal pole (approximately BA 8) and paracingulate gyrus (approximately BA 9 and 10). There were no regions identified in this analysis where activation was negatively correlated with the symptom index. We also had included the dysphoric/anxious index as a covariate within the ought vs. control contrast. However, we found no regions where there was significantly greater activation in response to ought vs. yoked-control priming as a function of negative affect.

**Table 3:** **Regions showing significantly activation positively correlated with symptom index scores following promotion (ideal) priming trials compared to control priming trials**.

**Probabilistic anatomical label**	***x***	***y***	***z***	***Z* statistic**	**Cluster volume (*p*)**
Frontal pole (36%), superior frontal gyrus (32%)	8	40	52	4.59	2295 mm^3^ (*p* < 0.01)
Paracingulate gyrus (57%), frontal pole (24%)	8	54	4	3.95	
Paracingulate gyrus (40%), anterior cingulate gyrus (13%)	–12	44	16	3.84	
Frontal pole (24%)	–12	56	42	3.74	
Frontal pole (21%)	26	56	36	3.71	
Frontal pole (14%)	–20	58	36	3.31	

Coordinates of local maxima within each cluster of activation are in MNI space. Probabilistic labels reflect the likelihood that a coordinate belongs to a given region; for clarity, only labels whose likelihood exceeds 5% are shown.

## Discussion

Despite their centrality for behavior, motivation, mood, and identity, only recently have personal goals such as hopes, dreams, and obligations been examined from a cognitive neuroscience perspective. Personal goals are similar to more concrete, situation-specific goals in that they frequently entail either approach or avoidance, but they are distinct with regard to their abstractness, their motivational significance, and their centrality to the self. In this study, we explored the neural correlates of two classes of personal goals: promotion goals, which represent desired outcomes that an individual would attain by “making good things happen,” and prevention goals, which also represent desired outcomes but which are attained by “keeping bad things from happening.” Using rapid masked priming with idiographically selected ideal and ought goals (both of which are desired personal attributes), we observed distinct neural activation patterns for the two goal types. In addition, we found that activation following goal priming is modulated by individual differences in perceived success of promotion and prevention goal attainment (but not BAS/BIS strength) as well as by current level of negative affect. Promotion and prevention goals have distinct neural correlates, in keeping with the behavioral distinctions between the two hypothesized motivational systems.

The activation patterns from the ideal > control and ought > control contrasts were reliably distinguishable and were most closely associated with the cortical midline structures model of the self (Northoff and Bermpohl, 2004). Nonetheless, it may be more accurate to say that priming of ideal and ought goals activated regions known to be associated with a key psychological process that RFT would predict is relevant: namely, the representation of desired outcomes for the self. We did not observe activation of regions most frequently associated with responses to spatiotemporal cues for either reward (e.g., ventral striatum) or threat (e.g., amygdala). Thus, the data are more consistent with a model organized primarily around personal goals as aspects of identity than a model of approach/avoidance or positive/negative affectivity. Of course, this could reflect the paradigm itself, particularly since our intent was to examine goal priming *per se* rather than a more extended cycle of ongoing self-evaluation triggered by the presence of a discrete cue for reward or threat.

We observed that promotion goal priming led to activation in frontal and occipital regions as well as caudate and thalamus, whereas prevention goal priming was associated with activation in precuneus and posterior cingulate cortex. Furthermore, individual differences in self-perceived success vs. failure to attain promotion and prevention goals were correlated with activation in specific regions that were differentially responsive to promotion vs. prevention goal priming. For those individuals with higher scores on the promotion success scale, the ideal > control contrast was associated with activation in additional regions including bilateral precuneus and both anterior and posterior cingulate cortex (proximal to, but not identical to, the loci observed for the main effect of the ought > control contrast). For participants scoring higher on the prevention success scale, ought goal priming was associated with activation in right occipital and parietal regions. Additionally, a correlation was observed between lower scores on the promotion success scale and left prefrontal activation following priming with ought goals.

The differences in activation observed for the two classes of personal goals were striking given that both sets of stimuli were self-generated by participants, both were self-descriptive, both were positively valenced, and both represented the kind of person the individual wanted to become. The only difference between the two stimulus sets was the kind of personal goal they represented—in one case (ideal), the individual's hopes, aspirations, and desired accomplishments, and in the other (ought), the individual's sense of duties, obligations, and responsibilities. In fact the same personal goal—for instance, to be successful—could be a promotion goal for one person and a prevention goal for another. RFT would predict that in such an instance, although the goal itself is identical, the motivational impetus, cognitive strategies, behavioral means, and affective responses to goal pursuit would differ radically. For the former individual, being successful would be attained by a strategic emphasis on accomplishment or being the best one could be, whereas for the latter, being successful would be attained by meeting one's responsibilities and obligations. Our findings provide evidence that the distinction between promotion and prevention goals is evident in terms of neural correlates from the moment such a goal is activated by a contextual cue.

The activation patterns associated with each type of personal goal involved regions within the cortical midline structures associated with self-referential processing as well as regions linked to other aspects of self-regulation (Amodio and Frith, 2006; Beer et al., 2009; Heatherton, 2011). For example, promotion goal priming-activated areas within orbital and medial PFC, both of which have been reliably demonstrated to be linked to a range of self-referential mental processes, including representation and monitoring of self-referential knowledge (Northoff and Bermpohl, 2004). Promotion priming also was associated with activation in the caudate and thalamus, which have been identified as components of both intuition and implicit learning as well as broader networks underlying reward sensitivity, preparatory motor functions, social judgment, and goal pursuit behavior (e.g., Lieberman, 2000; Rameson et al., 2010). The greater caudate activation associated with ideal priming among high-success individuals also may reflect the role of the caudate in reward learning and appetitive goal pursuit. Thus, ideals may engage regions that subserve representation and pursuit of abstract positive outcomes via direct, task-focused activity. In contrast, prevention goal priming was associated with activation in precuneus and posterior cingulate gyrus, which are implicated in the default mode network (e.g., Buckner et al., 2008) but also in phenomena such as self-reflection, self-awareness, and social adaptation (Pearson et al., 2011). As such, oughts may engage regions that support third-person perspective and moral reasoning.

Do the observed findings correspond to our knowledge of how individuals represent and pursue their goals? Given the likelihood that human psychological capabilities evolved in response to an increasingly complex social environment (Leary, 2004), activities such as the pursuit of personal goals would of necessity incorporate cognitive processes involving representations of self and significant others (Derryberry and Reed, 1996). In order for humans to survive and thrive, they must be not only capable of effective responses to survival-relevant stimuli. They also must be capable of representing and pursuing higher-order, cross-situational, socially embedded goals, and the representational, monitoring, and evaluative functions required must be integrated into coherent brain/behavior systems (Mischel, 2004). For the higher-order personal goals that people pursue, there is no necessarily spatiotemporal “moving toward or away from”; rather, the strategies people use involve “bringing about” or “making happen” (Carver and Scheier, 1998). This phenomenological distinction raises the possibility that brain/behavior systems for *strategic* approach and avoidance, such as are postulated in RFT, would be functionally discriminable from those for spatiotemporal approach and avoidance. For example, personal goals such as being successful, or intelligent, or trustworthy require a top-down coordination that brings relevant concrete goal representations into working memory across a range of situations (Carver and Scheier, 1990). The present data could be interpreted as reflecting how such top-down coordination is manifested differentially within the brain when promotion or prevention self-regulation is engaged.

Our findings were somewhat different from those reported by Eddington et al. (2007, 2009), especially in the case of promotion priming, where left orbital prefrontal activation was found to be discriminantly associated with promotion goal cues which had been presented in the context of a self-reference judgment task. We see the distinctions between the two sets of findings as primarily reflecting differences in the experimental paradigm used. Eddington et al. (2007, 2009) used a task that had been designed to identify neural signatures of explicit self-referential processing; they simply included a set of idiographically selected ideal and ought attributes within the stimuli used for the judgment task. Their findings, in essence, revealed a promotion-goal-specific activation pattern which was embedded within an instructional set emphasizing self-descriptiveness and requiring a specific judgment. In contrast, the rapid masked priming paradigm in the present study was selected so that goal-related activation patterns could be observed without the potentially complicating factor of an explicit self-evaluation in reference to the goal being primed. The distinction between the activation patterns found by Eddington and colleagues and those observed in the present study warrants additional study.

Based on these findings, does RFT help to refine our knowledge of the brain regions involved in the representation of personal goals? We suggest that in terms of the postulated distinctions between promotion and prevention, the answer is a qualified yes. The psychological dynamics of the promotion system can be viewed in signal detection terms (Tanner and Swets, 1954; Trope and Liberman, 1996), particularly as involving eagerness, which increases with greater proximity (in this case, conceptually or symbolically rather than spatiotemporally) to the target (Higgins, 1998). As such, we postulate that self-reflection may be less central to promotion-based goal pursuit, since promotion goals “loom larger” as the individual gets closer to attaining them (Higgins, 1998), and indeed we did not observe main effects of promotion-triggered activation in posterior midline structures associated with self-reflective thought.

Prevention system dynamics also can be viewed in signal detection terms, with the system organized to avoid errors of commission (albeit still in the service of ultimately attaining a positive end-state). As such, the prevention system relies upon vigilance as the dominant motivational state and self-evaluation as a recursive cognitive process. Consistent with this characterization, prevention goal priming was associated with activation in areas engaged by tasks that require self-reflection and even self-awareness, as well as by decisions involving judgments of morality and principle (e.g., Greene and Haidt, 2002). Such processes are of primary relevance to the pursuit of personal goals that are construed in terms of obligation, responsibility, or a sense of “should” (Higgins, 1997).

Of course, the findings do not represent a self-contained set of brain/behavior systems associated with personal goal pursuit. Self-regulation is too complex and multifaceted to be modeled adequately by a single experimental task. It would have been unlikely to find activation in areas associated with psychological processes that are substantially “downstream” from goal activation, such as consummatory behavior, goal disengagement, or affect regulation. Nonetheless, the activation patterns immediately following promotion and prevention goal priming were discriminable, and the rapid masked priming technique provided sufficient sensitivity to detect those patterns—which appeared to be broadly consistent with RFT's conceptualizations for each system.

We also observed that individual differences in self-reported success pursuing a particular kind of goal predicted activation following priming with idiographic exemplars of such goals, whereas individual differences in temperament-based approach and avoidance tendencies (operationalized using the Carver and White BIS/BAS Scales) did not. The behavioral activation and inhibition systems are hypothesized to represent inborn, presumably genetically determined variation in sensitivity to cues for spatiotemporal approach/avoidance behavior and/or the intensity and duration of behavioral responses to such cues. In contrast, individual differences in the strength of the promotion and prevention systems have been linked to socialization (Manian et al., 2006), and in particular to variability in the messages parents convey to their children about the relative importance of making good things happen vs. keeping bad things from happening. Strauman and Wilson (2010) postulated that the two sets of systems have different phylogenetic and developmental origins as well as distinguishable functions. Whereas BAS and BIS appear to operate in response to immediate, concrete cues for reward and danger respectively, promotion and prevention operate as “world views” or cognitive styles and provide a functional link between pursuit of higher-order personal goals and the social world. Eddington et al. (2007) reported that the Carver and White scales did not predict neural responses to promotion or prevention goal priming, and the present data are in concordance with their findings. Together, the two studies provide evidence that the neurobiological bases of BAS/BIS and promotion/prevention are likely to be distinct—and offer the possibility that the two sets of systems interact in complex ways to influence goal-directed behavior in any specific situation.

Both RFT and its precursor, self-discrepancy theory (Higgins, 1987), predict that perceived lack of progress toward a motivationally significant personal goal would be associated with negative affect. Both theories also postulate that chronic distress, as well as emotional disorders such as depression and anxiety, can interfere with effective goal pursuit (Strauman, 2002). To explore the impact of negative affect on personal goal activation, we examined whether individual variability in negative affect (assessed by creating a dysphoric/anxious index from the BDI and STAI) would predict neural responses to promotion or prevention goal priming. For individuals reporting greater levels of distress, promotion priming was associated with additional activation in the bilateral frontal pole and paracingulate gyrus. Prevention goal priming did not reveal any regions where activation was modulated by self-reported distress.

What might this pattern of results signify about the influence of chronic distress on self-regulation? Level of negative affect was associated with greater recruitment of bilateral frontal regions in response to cues for “making good things happen,” suggesting that higher levels of distress could introduce a greater degree of recruitment of prefrontal regions in the goal pursuit process. More speculatively, such increased frontal activity could signify activation of negative cognitive schemas, as hypothesized in cognitive models of depression (e.g., Beck et al., 1979), which would be likely to interfere with effective strategic pursuit of promotion goals. The nature of the promotion system dictates that the individual's optimal strategy is to ignore errors or unsuccessful trials and continue with goal pursuit efforts. Too much engagement of self-evaluation would complicate goal pursuit efforts by diverting processing resources away from the eager motivational state that characterizes successful promotion goal pursuit. The study was not intended to explore clinically relevant emotional states and symptom patterns, and it may be premature to extrapolate these findings to depression and related internalizing disorders. Still, the modulation of neural response to ideal priming by negative affect was robust enough to emerge from an analysis that included other statistically significant covariates.

In summary, the rapid masked stimulus presentation technique that we used to prime participants' promotion and prevention goals led to interpretable and reliably distinguishable patterns of neural activation. The findings are broadly consistent with established findings from the social neuroscience literature, and the discriminability of the activation patterns associated with the two types of personal goals provides support for the critical distinction in RFT between promotion and prevention as modes of self-regulation. To the extent that those modes become automatic and systematized over time, then perhaps it will be of value to speak of promotion and prevention “systems” assuming that present data are replicated using other goal-relevant tasks. Furthermore, to the extent that individual differences relevant to self-regulation of personal goal pursuit might be expected to influence the strength and accessibility of the two systems, our findings also were consistent with the view that the neural correlates of the systems may differ as a function of individuals' beliefs about their success vs. failure in attaining such goals.

We close with several comments about the limitations of the present research and its potential implications for future studies. As noted previously, the priming paradigm may be a reasonable operationalization of one phase of a self-regulatory cycle—automatic activation of goal pursuit via exposure to a goal-relevant cue—but it would not be likely to identify neural responses to other phases of that cycle, such as regulation of affect in response to goal pursuit feedback or other conscious processes. Likewise, we did not systematically choose the participants on the basis of potentially relevant individual differences, and the priming task did not explicitly target such individual variability. Our analyses of those individual differences (in regulatory focus, in BAS/BIS strength, and in chronic distress) are correlational and represent first steps in an ongoing process of investigation. It should be emphasized that the promotion and prevention systems are constructs, in the same way as more familiar brain/behavior systems. We are using those constructs to guide prediction and interpretation but not making claims about neural structure or neuroanatomical connectivity. Nonetheless, we offer these findings as, to our knowledge, the first neuroimaging evidence for the construct validity of the promotion and prevention systems and the critical roles that individual differences and chronic affective states play in the day-to-day function of those systems.

### Conflict of interest statement

The authors declare that the research was conducted in the absence of any commercial or financial relationships that could be construed as a potential conflict of interest.

## References

[B1] Allport G. W. (1955). Becoming: Basic Considerations for a Theory of Personality. New Haven, CT: Yale University Press

[B2] Amodio D. M. Frith C. D. (2006). Meeting of minds: the medial frontal cortex and social cognition. Nat. Neurosci. Rev. 7, 268–277 10.1038/nrn1884 16552413

[B3] Amodio D. M. Master S. L. Yee C. M. Taylor S. E. (2007). Neurocognitive components of the behavioral inhibition and activation systems: implications for theories of self-regulation. Psychophysiology 44, 1–9 10.1111/j.1469-8986.2007.00609.x 17910730

[B4] Amodio D. M. Shah J. Y. Sigelman J. Brazy P. C. Harmon-Jones E. (2004). Implicit regulatory focus associated with asymmetrical frontal cortical activity. J. Exp. Soc. Psychol. 40, 225–232

[B5] Austin J. T. Vancouver J. B. (1996). Goal constructs in psychology: structure, process, and content. Psychol. Bull. 120, 338–375

[B6] Beck A. T. Mendelson M. Mock J. Erbaugh J. (1961). An inventory for measuring depression. Arch. Gen. Psychiatry 4, 561–571 10.1001/archpsyc.1961.01710120031004 13688369

[B7] Beck A. T. Rush A. J. Shaw B. F. Emery G. (1979). Cognitive Therapy of Depression. New York, NY: Guilford Press

[B8] Beckmann C. F. Jenkinson M. Smith S. M. (2003). General multilevel linear modeling for group analysis in FMRI. Neuroimage 20, 1052–1063 10.1016/S1053-8119(03)00435-X 14568475

[B9] Beer J. S. Lombardo M. V. Bhanji J. P. (2009). Roles of medial prefrontal cortex and orbitofrontal cortex in self-evaluation. J. Cogn. Neurosci. 22, 2108–2119 10.1162/jocn.2009.21359 19925187 PMC4159715

[B10] Beer J. S. Ochsner K. N. (2006). Social cognition: a multi level analysis. Brain Res. 1079, 98–105 10.1016/j.brainres.2006.01.002 16513097

[B11] Bjork J. M. Knutson B. Fong G. W. Caggiano D. M. Bennett S. M. Hommer D. W. (2004). Incentive-elicited brain activation in adolescents: similarities and differences from young adults. J. Neurosci. 24, 1793–1802 10.1523/JNEUROSCI.4862-03.2004 14985419 PMC6730402

[B12] Bowlby J. (1988). A Secure Base: Clinical Applications of Attachment Theory. London: Routlege

[B13] Buckner R. L. Andrews-Hanna J. R. Schachter D. L. (2008). The brain's default network: anatomy, function, and relevance to disease. Ann. N.Y. Acad. Sci. 1124, 1–38 10.1196/annals.1440.011 18400922

[B14] Cantor N. Zirkel S. (1990). Personality, cognition, and purposive behavior, in Handbook of Personality: Theory and Research, ed Pervin L. A. (New York, NY: Guilford Press), 135–164

[B15] Carver C. S. Scheier M. F. (1990). Origins and functions of positive and negative affect: a control-process view. Psychol. Rev. 97, 19–35

[B16] Carver C. S. Scheier M. F. (1998). On the Self-regulation of Behavior. New York, NY: Cambridge University Press

[B17] Carver C. S. White T. L. (1994). Behavioral inhibition, behavioral activation, and affective responses to impending reward and punishment. The BIS/BAS Scales. J. Pers. Soc. Psychol. 67, 319–333

[B18] Clithero J. A. Reeck C. Carter R. M. Smith D. V. Huettel S. A. (2011). Nucleus accumbens mediates relative motivation for rewards in the absence of choice. Front. Hum. Neurosci. 5:87 10.3389/fnhum.2011.00087 21941472 PMC3171065

[B19] Conway M. A. (2001). Sensory perceptual episodic memory and its context: autobiographical memory. Philos. Trans. R. Soc. Lond. B Biol. Sci. 356, 1375–1384 10.1098/rstb.2001.0940 11571029 PMC1088521

[B20] Conway M. A. Pleydell-Pierce C. W. (2000). The construction of autobiographical memories in the self memory system. Psychol. Rev. 107, 261–288 10789197 10.1037/0033-295x.107.2.261

[B21] Cunningham W. A. Raye C. L. Johnson M. K. (2005). Neural correlates of evaluation associated with promotion and prevention regulatory focus. Cogn. Behav. Affect. Neurosci. 5, 202–211 16180626 10.3758/cabn.5.2.202

[B22] Davidson R. J. Irwin W. (1999). The functional neuroanatomy of emotion and affective style. Trends Cogn. Sci. 3, 11–21 10.1016/S1364-6613(98)01265-0 10234222

[B23] Dehaene S. Naccache L. Cohen L. LeBihan D. Mangin J. F. Poline J.-B. (2001). Cerebral mechanisms of word masking and unconscious repetition priming. Nat. Neurosci. 4, 752–758 10.1038/89551 11426233

[B24] Derryberry D. Reed M. A. (1996). Regulatory processes and the development of cognitive representations. Dev. Psychopathol. 8, 215–234

[B25] Diaz M. T. McCarthy G. (2007). Unconscious word processing engages a distributed network of brain regions. J. Cogn. Neurosci. 19, 1768–1775 10.1162/jocn.2007.19.11.1768 17958480

[B26] Eddington K. M. Dolcos F. Cabeza R. Krishnan K. R. R. Strauman T. J. (2007). Neural correlates of promotion and prevention goal activation: an fMRI study using an idiographic approach. J. Cogn. Neurosci. 19, 1152–1162 10.1162/jocn.2007.19.7.1152 17583991

[B27] Eddington K. M. Dolcos F. McLean A. N. Cabeza R. Krishnan K. R. R. Strauman T. J. (2009). Neural correlates of idiographic goal priming in depression: goal-specific dysfunctions in the orbitofrontal cortex. Soc. Cogn. Affect. Neurosci. 4, 238–246 10.1093/scan/nsp016 19433416 PMC2728635

[B27a] Gray J. A. (1990). Brain systems that mediate both emotion and cognition. Cogn. Emotion 4, 269–288

[B28] Gray J. A. (1994). Three fundamental emotion systems, in The Nature of Emotion, eds Ekman P. Davidson R. J. (New York, NY: Oxford University Press), 243–247

[B29] Greene J. Haidt J. (2002). How (and where) does moral judgment work? Trends Cogn. Sci. 6, 517–527 10.1016/S1364-6613(02)02011-9 12475712

[B30] Heatherton T. F. (2011). Neuroscience of self and self-regulation. Annu. Rev. Psychol. 62, 363–390 10.1146/annurev.psych.121208.131616 21126181 PMC3056504

[B31] Higgins E. T. (1987). Self-discrepancy: a theory relating self and affect. Psychol. Rev. 94, 319–340 3615707

[B32] Higgins E. T. (1997). Beyond pleasure and pain. Am. Psychol. 52, 1280–1300 9414606 10.1037//0003-066x.52.12.1280

[B33] Higgins E. T. (1998). Promotion and prevention: regulatory focus as a motivational principle, in Advances in Experimental Social Psychology, Vol. 30, ed Zanna M. P. (New York, NY: Academic Press), 1–46

[B34] Higgins E. T. (2012). Beyond Pleasure and Pain. How Motivation Works. New York, NY: Oxford University Press

[B35] Higgins E. T. Bond R. N. Klein R. Strauman T. (1986). Self-discrepancies and emotional vulnerability: how magnitude, accessibility, and type of discrepancy influence affect. J. Pers. Soc. Psychol. 51, 5–15 3735070 10.1037/0022-3514.51.1.5

[B36] Higgins E. T. Friedman R. S. Harlow R. E. Idson L. Ayduk O. Taylor A. (2001). Achievement orientations from subjective histories of success: promotion pride versus prevention pride. Eur. J. Soc. Psychol. 31, 3–23

[B37] James W. (1948). The Principles of Psychology. New York, NY: World Publishing (Original work published 1890).

[B38] Kelly G. A. (1955). The Psychology of Personal Constructs. New York, NY: W. W. Norton

[B39] Kringelbach M. L. (2005). The human orbitofrontal cortex: linking reward to hedonic experience. Nat. Rev. Neurosci. 6, 691–702 10.1038/nrn1747 16136173

[B40] Leary M. R. (2004). The sociometer, self-esteem, and the regulation of interpersonal behavior, in Handbook of Self-regulation: Research, Theory, and Applications, eds Vohs K. Baumeister R. (New York, NY: Guilford Press), 373–391

[B41] Lieberman M. D. (2000). Intuition: a social cognitive neuroscience approach. Psychol. Bull. 126, 109–137 10668352 10.1037/0033-2909.126.1.109

[B42] Lou H. C. Luber B. Stanford A. Lisanby S. H. (2010). Self-specific processing in the default network: a single-pulse TMS study. Exp. Brain Res. 207, 27–38 10.1007/s00221-010-2425-x 20878395 PMC3008414

[B43] Manian N. Papadakis A. M. Strauman T. J. Essex M. (2006). The development of children's ideal and ought self-guides: the influence of parenting on individual differences in guide strength. J. Pers. 74, 1619–1645 10.1111/j.1467-6494.2006.00422.x 17083660

[B44] McClure S. M. York M. K. Montague P. R. (2004). The neural substrates of reward processing in humans: the modern role of fMRI. Neuroscientist 10, 260–268 10.1177/1073858404263526 15155064

[B45] Mischel W. (2004). Toward an integrative science of the person. Annu. Rev. Psychol. 55, 1–22 10.1146/annurev.psych.55.042902.130709 14744208

[B46] Morf C. C. Mischel W. (2012). The self as a psycho-social dynamic processing system: toward a converging science of selfhood, in Handbook of Self, and Identity, 2nd Edn eds Leary M. R. Tangney J. P. (New York, NY: Guilford Press), 21–49

[B47] Nitschke J. B. Heller W. Imig J. McDonald R. P. Miller G. A. (2001). Distinguishing dimensions of anxiety and depression. Cognit. Ther. Res. 25, 1–22 10.1016/j.janxdis.2011.02.001 21377316 PMC3074026

[B48] Northoff G. Bermpohl F. (2004). Cortical midline structures and the self. Trends Cogn. Sci. 8, 102–107 10.1016/j.tics.2004.01.004 15301749

[B49] Packer D. J. Cunningham W. A. (2009). Neural correlates of reflection on goal states: the role of regulatory focus and temporal distance. Soc. Neurosci. 4, 412–425 10.1080/17470910902750186 19739033

[B50] Pearson J. M. Heilbronner S. R. Barack D. L. Hayden B. Y. Platt M. L. (2011). Posterior cingulate cortex: adapting behavior to a changing world. Trends Cogn. Sci. 15, 143–149 10.1016/j.tics.2011.02.002 21420893 PMC3070780

[B51] Qin P. Northoff G. (2011). How is our self related to midline regions and the default-mode network? Neuroimage 57, 1221–1233 10.1016/j.neuroimage.2011.05.028 21609772

[B52] Rameson L. T. Satpute A. B. Lieberman M. D. (2010). The neural correlates of implicit and explicit self-relevant processing. Neuroimage 50, 701–708 10.1016/j.neuroimage.2009.12.098 20045472

[B53] Reuter M. Stark R. Hennig J. Walter B. Kirsch P. Schienle A. (2004). Personality and emotion: test of Gray's personality theory by means of an fMRI study. Behav. Neurosci. 118, 462–469 10.1037/0735-7044.118.3.462 15174923

[B54] Rogers C. R. (1961). On Becoming a Person. Boston, MA: Houghton-Mifflin

[B55] Smith S. M. Jenkinson M. Woolrich M. W. Beckmann C. F. Behrens T. E. Johansen-Berg H. (2004). Advances in functional and structural MR image analysis and implementation as FSL. Neuroimage 23 Suppl. 1, S208–S219 15501092 10.1016/j.neuroimage.2004.07.051

[B56] Spielberger C. D. Gorsuch R. L. Lushene R. E. (1970). State-trait Anxiety Inventory. Palo Alto, CA: Consulting Psychologists Press

[B57] Strauman T. J. (1990). Self-guides and emotionally significant childhood memories: a study of retrieval efficiency and negative emotional content. J. Pers. Soc. Psychol. 59, 869–880

[B58] Strauman T. J. (1996). Stability within the self: a longitudinal study of the implications of self-discrepancy theory. J. Pers. Soc. Psychol. 71, 1142–1153 8979383 10.1037//0022-3514.71.6.1142

[B59] Strauman T. J. (2002). Self-regulation and depression. Self Identity 1, 151–157

[B60] Strauman T. J. Wilson W. A. (2010). Individual differences in approach and avoidance: behavioral activation/inhibition and regulatory focus as distinct systems, in Handbook of Self-Regulation and Personality, ed Hoyle R. (New York, NY: Guilford Press), 447–473

[B61] Sullivan H. S. (1953). The interpersonal theory of psychiatry, in The Collected Works of Harry Stack Sullivan (Vol. 1), eds Perry H. S. Gawel M. L. (New York, NY: W. W. Norton and Co.), 10–48

[B62] Tanner W. P. Jr. Swets J. A. (1954). A decision making-theory of visual detection. Psychol. Rev. 61, 401–409 13215690 10.1037/h0058700

[B63] Touryan S. R. Johnson M. K. Mitchell K. J. Farb N. Cunningham W. A. Raye C. L. (2007). The influence of self-regulatory focus on encoding of, and memory for, emotional words. Soc. Neurosci. 2, 14–27 10.1080/17470910601046829 18633804

[B64] Trope Y. Liberman A. (1996). Social hypothesis testing: cognitive and motivational mechanisms, in Social psychology: Handbook of basic principles, eds Higgins E. T. Kruglanski A. W. (New York, NY: Guilford Press), 239–270

[B65] Watson D. Wiese D. Vaidya J. Tellegen A. (1999). The two general activation systems of affect: structural findings, evolutionary considerations, and psychobiological evidence. J. Pers. Soc. Psychol. 76, 820–838

[B66] Woolrich M. W. Behrens T. E. Beckmann C. F. Jenkinson M. Smith S. M. (2004). Multilevel linear modeling for fmri group analysis using bayesian inference. Neuroimage 21, 1732–1747 10.1016/j.neuroimage.2003.12.023 15050594

[B67] Woolrich M. W. Jbabdi S. Patenaude B. Chappell M. Makni S. Behrens T. (2009). Bayesian analysis of neuroimaging data in FSL. Neuroimage 45 Suppl. 1, S173–S186 10.1016/j.neuroimage.2008.10.055 19059349

[B68] Woolrich M. W. Ripley B. D. Brady M. Smith S. M. (2001). Temporal autocorrelation in univariate linear modeling of Fmri data. Neuroimage 14, 1370–1386 10.1006/nimg.2001.0931 11707093

[B69] Worsley K. J. (2001). Statistical analysis of activation images, in Functional Mri: An Introduction to Methods, eds Jezzard P. Matthews P. M. Smith S. M. (USA: Oxford University Press), 251–270

[B70] Zilles K. Amunts K. (2010). Centenary of Brodmann's Map – conception and fate. Nat. Rev. Neurosci. 11, 139–145 10.1038/nrn2776 20046193

